# Supplementary material for: Cyclin B1 scaffolds MAD1 at the kinetochore corona to activate the mitotic checkpoint
Source: EMBO J. 2020 Mar 23;39(12):e103180. doi: 10.15252/embj.2019103180 (PMC7298293; doi:10.15252/embj.2019103180)
Supplement: Supplementary file 1 — Appendix [file EMBJ-39-e103180-s001.pdf]

APPENDIX FOR

Cyclin B1 scaffolds MAD1 at the kinetochore corona to activate the mitotic checkpoint

Lindsey A Allan[1], Magda Reis[1], Giuseppe Ciossani[2], Pim J Huis in ’t Veld[2], Sabine Wohlgemuth[2], Geert JPL Kops[3], Andrea Musacchio[2] and Adrian T Saurin[1]

CONTENTS:

- APPENDIX FIGURE S1
- APPENDIX FIGURE S1 LEGEND

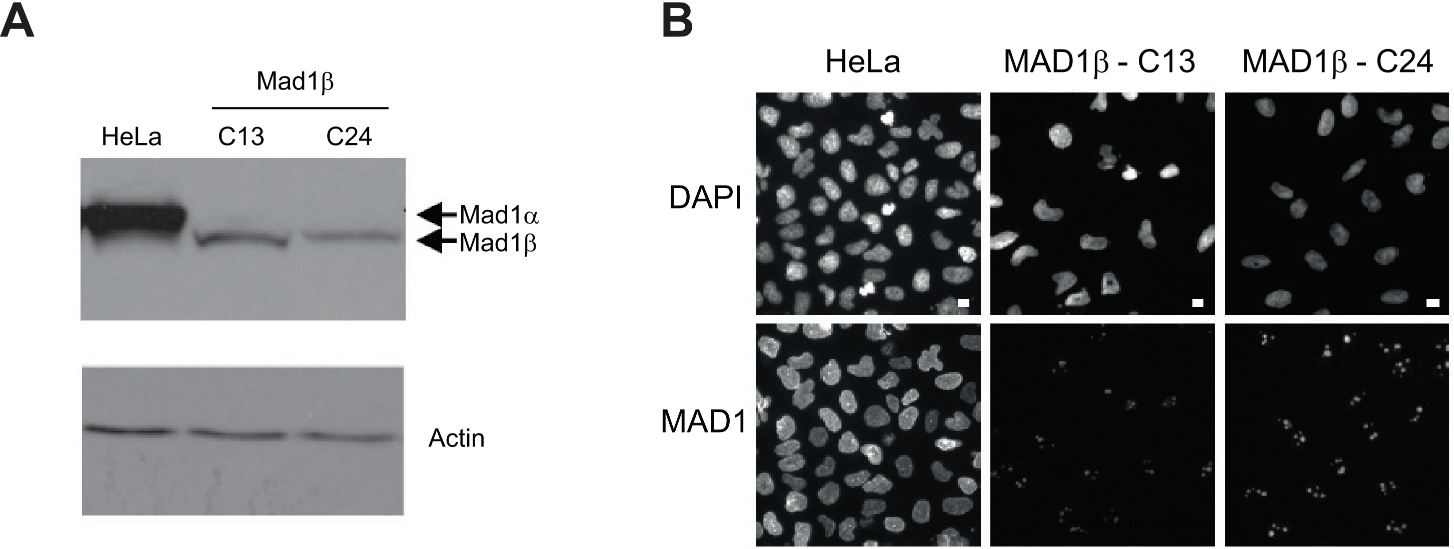

Figure S1. Validation of Mad1α knockout cell clones. A,B. Western blot (A) and immunofluorescence images (B) of MAD1 in control on MAD1α knockout HeLa cells (2 clones: MAD1α-C13 and C24). The lack of nuclear staining demonstrates a MAD1α knockout because MAD1β lacks a key NLS and is therefore cytoplasmic (Sze et al, 2008). Note, the cytoplasmic signal is not visible because immunofluorescence was performed on cells that were pre-extracted in 0.1% triton buffer (see methods).
