# Supplementary material for: Cyclin B1 scaffolds MAD1 at the kinetochore corona to activate the mitotic checkpoint
Source: EMBO J. 2020 Mar 23;39(12):e103180. doi: 10.15252/embj.2019103180 (PMC7298293; doi:10.15252/embj.2019103180)
Supplement: Supplementary file 2 — Expanded View Figures PDF [file EMBJ-39-e103180-s002.pdf]

Expanded View Figures

Figure EV1. Cyclin B1, not CDK1, interacts with the MAD1:MAD2 complex via the MAD1 N-terminus.

A–F Elution profiles and SDS–PAGE for SEC runs on the indicated columns of (A) CDK1 (blue), MBP-MAD1$^{1-92}$-SNAP (red; note that the same elution profile and SDS–PAGE are also displayed as reference in panel (B) and in Fig 2E to improve clarity) and their combination (green); (B) Cyclin B1 (blue), MBP-MAD1$^{1-92}$-SNAP (red) and their combination (green). Asterisks in (A and B) indicate free MBP, a byproduct of the MBP-MAD1$^{1-92}$-SNAP purification; (C) Cyclin B1:CDK1 (blue; note that the same elution profile and SDS–PAGE are also displayed as reference in panel (D) and in Fig 2E to improve clarity), MBP-MAD1$^{41-92}$ (red) and their combination (green); (D) Cyclin B1:CDK1 (blue), MBP-MAD1$^{60-92}$ (red) and their combination (green); (E) Cyclin B1:CDK1 (blue), MBP-MAD1$^{41-62}$ (red; note that the same elution profile and SDS–PAGE are also displayed as reference in panel F to improve clarity) and their combination (green). (F) Cyclin B1 (blue), MBP-MAD1$^{41-62}$ (red) and their combination (green). In all experiments, proteins were combined at 5 μM concentration.

© 2020 The Authors

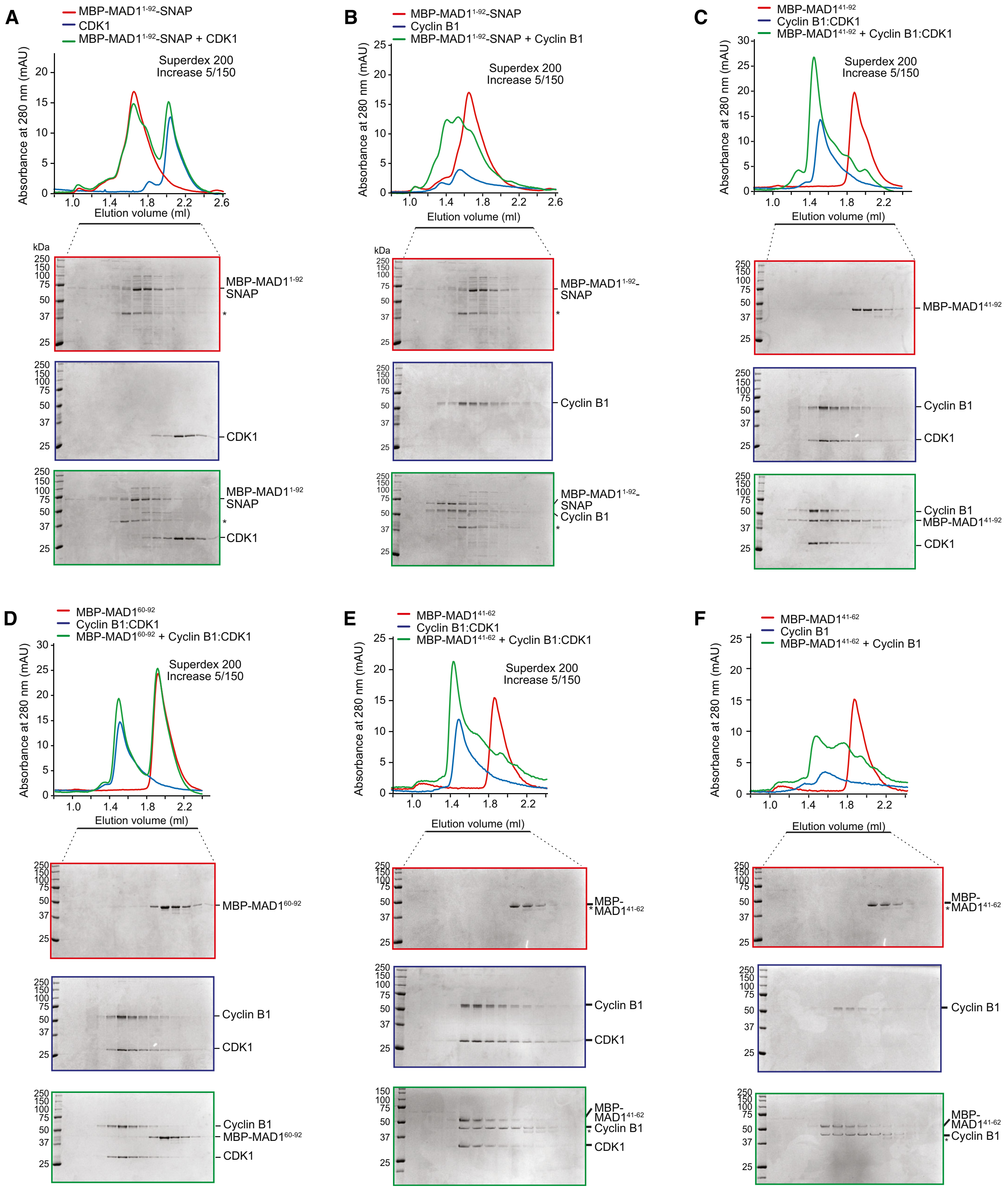

Figure EV1.

 © 2020 The Authors

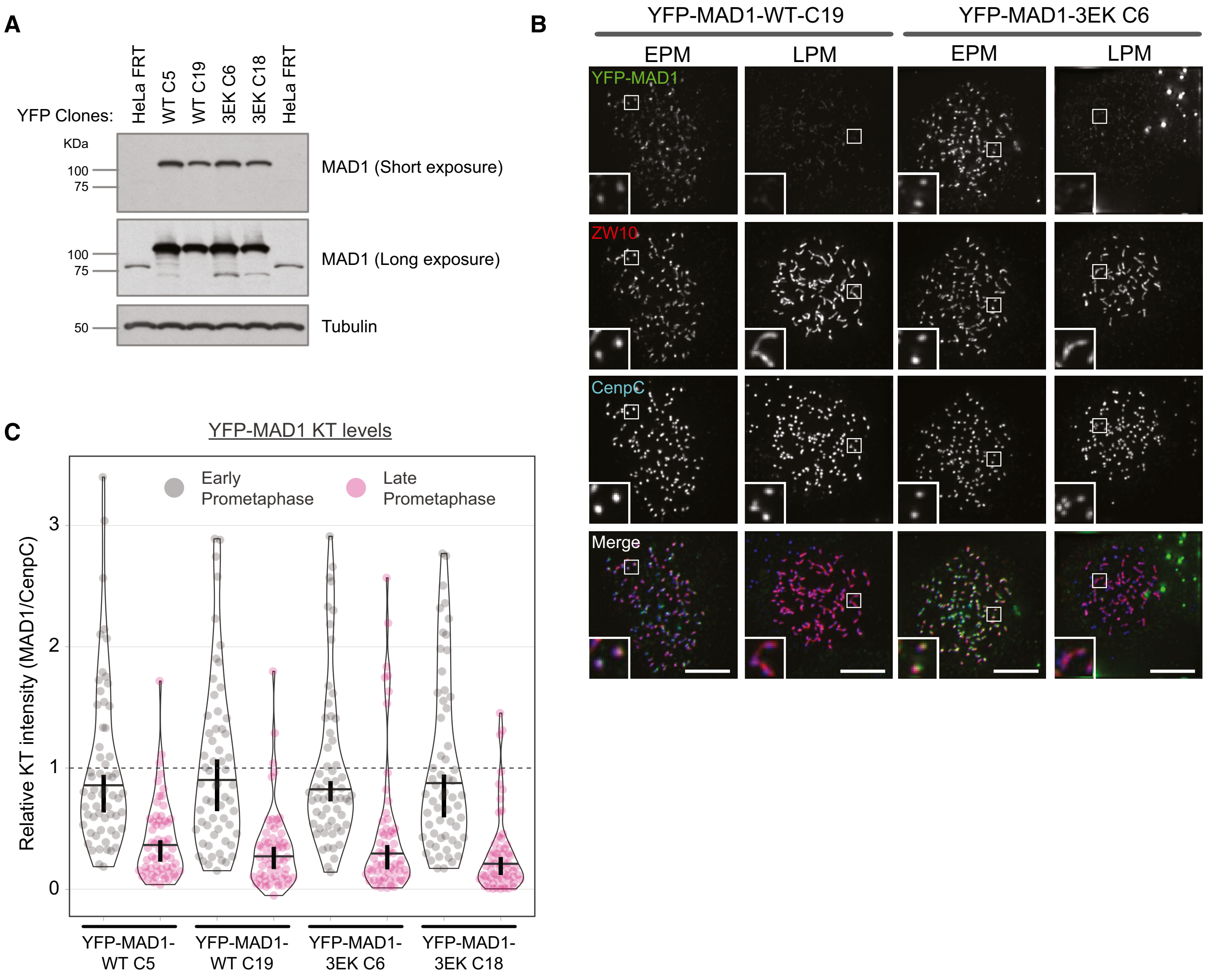

Figure EV2. YFP-MAD1-WT and YFP-MAD1-3EK do not localise to the corona.

A Western blot analysis of indicated YFP-MAD1-WT and YFP-MAD1-3EK clones treated with doxycycline.

B Immunofluorescence images showing MAD1 and ZW10 kinetochore levels in nocodazole-arrested YFP-MAD1-WT-C19 and 3EK-C6 cells just after nuclear envelope breakdown (early prometaphase—EPM) or later in mitosis when the chromatin is condensed (late prometaphase—LPM). Scale bars = 5 μM.

C Quantification of MAD1 kinetochore localisation from cells treated as in (B). In all kinetochore intensity graphs, each dot represents a cell, horizontal lines indicate the median, and vertical bars show the 95% confidence interval. Note, when these vertical bars do not overlap, the difference is considered statistically significant at a level of at least $P < 0.05$ (see Materials and Methods). The graph displays data that are relative to the early prometaphase controls, which are normalised to 1. The mean level of the normalised controls is indicated by the dotted lines. Sixty cells from 3 experiments.

© 2020 The Authors

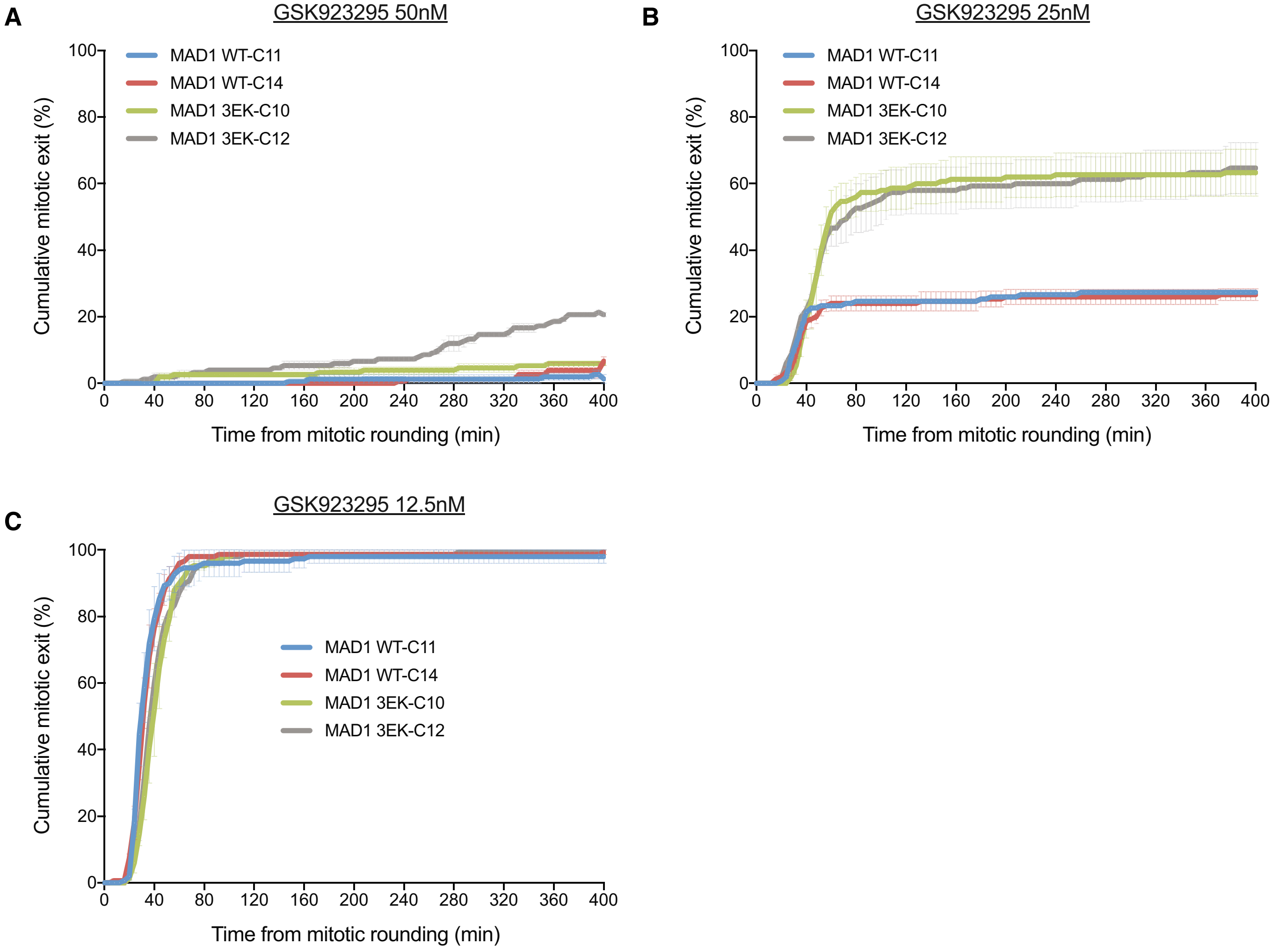

Figure EV3. MAD1-3EK cells arrest less efficiently following CENP-E inhibition.

A–C Duration of mitotic arrest in MAD1-WT and MAD1-3EK clones treated with 50 nM (A), 25 nM (B) or 12.5 nM (C) GSK923295 to inhibit CENP-E. Graph shows cumulative mean (±SEM) of 3 experiments, 50 cells per condition per experiment.

 © 2020 The Authors

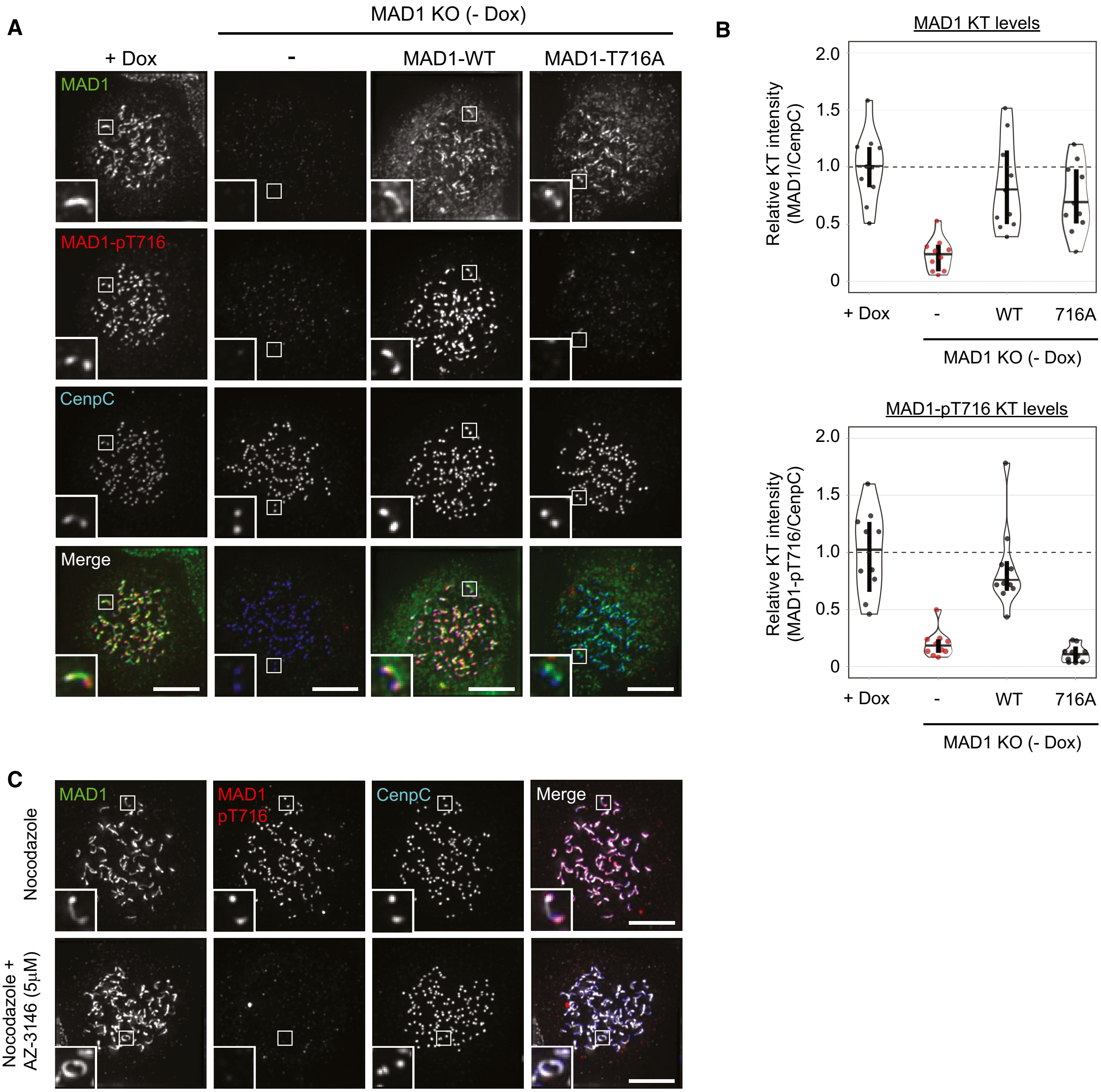

Figure EV4. Validating the MAD1-pT716 antibody in cells.

A, B Immunofluorescence images (A) and quantification (B) of relative MAD1 and MAD1-pT716 kinetochore levels in dox-inducible vsv-MAD1-WT cells with doxycycline removed (-Dox) and vsv-MAD1-WT or vsv-MAD1-T716A. Vsv-MAD1-WT cells were cultured without Dox for 11d prior to transient transfection with vsv-MAD1-WT or vsv-MAD1-T716A plasmids. After 43 h, nocodazole was added for 8 h prior to fixation. In all kinetochore intensity graphs, each dot represents a cell, horizontal lines indicate the median, and vertical bars show the 95% confidence interval. Note, when these vertical bars do not overlap, the difference is considered statistically significant at a level of at least $P < 0.05$ (see Materials and Methods). The graphs display data that are relative to doxycycline(dox)-treated controls, which are normalised to 1. The mean level of the normalised controls is indicated by the dotted lines.

C Immunofluorescence images of MAD1 and MAD1-pT716 kinetochore levels in nocodazole-arrested HeLa FRT cells.

Data information: Scale bars = 5 μM.

© 2020 The Authors

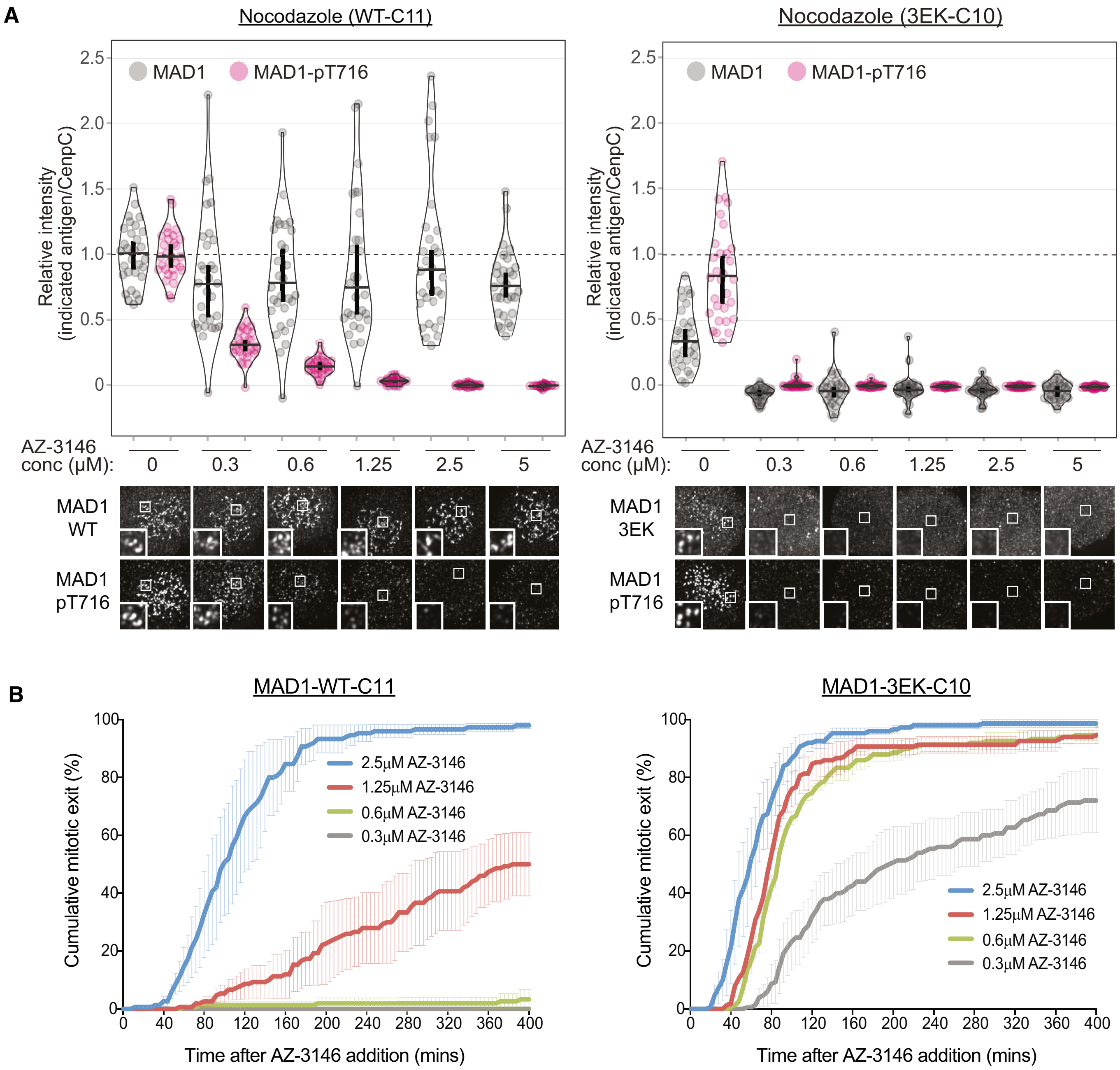

Figure EV5. Corona MAD1 allows the SAC to tolerate MPS1 inhibition by preserving MAD1 at kinetochores and enhancing MAD1-pT716 levels.

A Quantifications (top) and corresponding immunofluorescence images (underneath) of kinetochore MAD1 and MAD1-pT716 levels in nocodazole-arrested MAD1-WT-C11 and 3EK-C10 treated with different doses of AZ-3146 for 30 min. MG132 was included at the time of AZ-3146 addition to prevent mitotic exit. Each dot represents a cell, horizontal lines indicate the median and error bars show 95% confidence interval. Note, when these vertical bars do not overlap, the difference is considered statistically significant at a level of at least $P < 0.05$ (see Materials and Methods). Both kinetochore intensity graphs display data that are relative to the WT-C11 untreated controls, which are normalised to 1. The mean level of the normalised controls is indicated by the dotted lines. Thirty cells from 3 experiments.

B Duration of mitotic arrest in MAD1-WT-C11 or MAD1-3EK-C10 cells arrested in nocodazole and then treated with indicated concentrations of AZ-3146. Graph shows cumulative mean (±SEM) of 3 experiments, 50 cells per condition per experiment. Note, the 1.25 µM AZ-3146 data are displayed in Fig 4D, but also included here to allow comparison with other drug doses.

© 2020 The Authors
