# Supplementary material for: Cyclin B1 scaffolds MAD1 at the kinetochore corona to activate the mitotic checkpoint
Source: EMBO J. 2020 Mar 23;39(12):e103180. doi: 10.15252/embj.2019103180 (PMC7298293; doi:10.15252/embj.2019103180)
Supplement: Supplementary file 4 — Movie EV1 [file EMBJ-39-e103180-s004.zip › Movie EV1 legend.docx]

Movie EV1: Endogenous Cyclin B1-EYFP localisation during mitosis in RPE-1 cells
